# Why do we differ in number sense? Evidence from a genetically sensitive investigation^☆^

**DOI:** 10.1016/j.intell.2013.12.007

**Published:** 2014-03

**Authors:** M.G. Tosto, S.A. Petrill, J. Halberda, M. Trzaskowski, T.N. Tikhomirova, O.Y. Bogdanova, R. Ly, J.B. Wilmer, D.Q. Naiman, L. Germine, R. Plomin, Y. Kovas

**Affiliations:** a Department of Psychology, University of York, Heslington, York YO10 5DD, United Kingdom; b Department of Psychology, Tomsk State University, 36 Lenin Prospekt, 634050, Tomsk, Russia; c The Ohio State University, Columbus, OH 43210, United States; d Department of Psychological and Brain Sciences, The Johns Hopkins University, Baltimore, MD 21218-268, United States; e King's College London, MRC Social, Genetic and Developmental Psychiatry Centre, Institute of Psychiatry, De Crespigny Park, London SE5 8AF, United Kingdom; f Department of Psychology, Wellesley College, Central Street, Wellesley, MA 02481, United States; g Center for Human Genetic Research, Massachusetts General Hospital, Harvard Medical School, Boston, MA 02114, United States; h Goldsmiths, University of London, London SE14 6NW, United Kingdom

**Keywords:** Number sense, Mathematical ability, Behaviour genetics, Heritability, Directional selection

## Abstract

Basic intellectual abilities of quantity and numerosity estimation have been detected across animal species. Such abilities are referred to as ‘number sense’. For human species, individual differences in number sense are detectable early in life, persist in later development, and relate to general intelligence. The origins of these individual differences are unknown. To address this question, we conducted the first large-scale genetically sensitive investigation of number sense, assessing numerosity discrimination abilities in 837 pairs of monozygotic and 1422 pairs of dizygotic 16-year-old twin pairs. Univariate genetic analysis of the twin data revealed that number sense is modestly heritable (32%), with individual differences being largely explained by non-shared environmental influences (68%) and no contribution from shared environmental factors. Sex-Limitation model fitting revealed no differences between males and females in the etiology of individual differences in number sense abilities. We also carried out *Genome-wide Complex Trait Analysis* (GCTA) that estimates the population variance explained by additive effects of DNA differences among unrelated individuals. For 1118 unrelated individuals in our sample with genotyping information on 1.7 million DNA markers, GCTA estimated zero heritability for number sense, unlike other cognitive abilities in the same twin study where the GCTA heritability estimates were about 25%. The low heritability of number sense, observed in this study, is consistent with the directional selection explanation whereby additive genetic variance for evolutionary important traits is reduced.

## Highlights

• Modest heritability of numerosity discrimination skills at 16 years
• GCTA analysis supports directional selection for numerosity discrimination skills.
• No sex differences in the etiology of individual variation in numerosity at age 16

## 1 Introduction

Numbers, in their symbolic notation, form a basic tally system to answer the questions of ‘how much’ or ‘how many’. Numerals are an efficient way to keep track of discrete quantities and numerosities. This is particularly useful if the numerosities to be represented are relatively large. An alternative way to represent quantities and numerosities is to evaluate them in terms of ‘more’ or ‘less’; this approach does not require the use of symbols or any learned system and is based on approximation. The mechanism supporting such approximations, the approximate number system, is also often referred to as ‘number sense’ (see Dehaene, 1997 for a review). The exact definition and measurement of number sense are often debated (see Berch, 2005). This paper will refer to number sense as an intellectual ability that allows us to represent, estimate and manipulate non-symbolic quantities/numerosities. A practical example of using number sense is when, without counting and after a quick glance, we join the queue with the fewest people.

Number sense has attracted considerable attention as individual differences in this ability have been found to be associated with mathematical ability (e.g. Jordan, Kaplan, Oláh, & Locuniak, 2006).

One of the theories underlying mathematical learning is that numeracy skills partially originate from non-symbolic numerosity ability interfacing with the taught symbolic numerical system (e.g. Dehaene, 1997; Feigenson, Dehaene, & Spelke, 2004; Izard, Pica, Spelke, & Dehaene, 2008). It has been proposed that deficits in manipulating numerosity are one of the signatures of mathematical difficulties (Butterworth, 1999, 2010; Landerl, Bevan, & Butterworth, 2004; Mazzocco, Feigenson, & Halberda, 2011). There is evidence that symbolic (dealing with numerals) and non-symbolic (dealing with numerosity) number systems contribute interactively to the development of normal arithmetic skills. For example, the native language of a small Amazonian tribe, the Mundurukú, has words for numbers only up to five. Although Mundurukú participants can approximate quantities well above their naming range, they fail to manipulate exact numbers. This indicates that the approximate number system is independent from the verbal encoding of numbers that produces exact numerical representations. Further, if the non-symbolic quantities fail to map onto their symbolic correspondence, the emergence of exact arithmetic may not typically develop (Pica, Lemer, Izard, & Dehaene, 2004).

Some studies, however, challenge the view of a significant relationship between symbolic and non-symbolic representation of numbers. In one study, mathematical achievement in 6-to 7-year-old children correlated with Numerical Distance Effect (speed and accuracy in number comparison are greater when the numerical distance separating two numbers is relatively large, i.e. 3 and 9 vs 3 and 5) in symbolic, but not in non-symbolic comparisons (Holloway & Ansari, 2009). Similarly, children with mathematical disabilities show impairments in comparisons of number symbols, but not in the processing of non-symbolic numerical magnitudes (Rousselle & Noël, 2007).

### 1.1 Numerosity discrimination in animals and humans

The approximate number system is not unique to humans. Many animal species can approximate numerosities and can remember discrete number of objects and events. Basic numerical competences have been reported in social and non-social animals (ants: Reznikova & Ryabko, 2011; bears: Vonk & Beran, 2012); mosquito fish discriminate quantities using numerical cues and can be trained to recognize a set of two items from another with three (Agrillo, Dadda, Serena, & Bisazza, 2009; Agrillo, Piffer, & Bisazza, 2011); and rats can distinguish between arrays with different numbers of auditory signals (Meck & Church, 1983). In addition to estimation abilities, rudimentary arithmetic skills performed on numerosity sets (i.e. collection of discrete items) have been reported by studies that used attachment paradigms with newborn chicks (Rugani, Fontanari, Simoni, Regolin, & Vallortigara, 2009; Rugani, Regolin, & Vallortigara, 2011).

Animal evidence suggests that basic numerical competences are independent from language and are present at birth. Studies of human infants also show that this ability is preverbal. Using habituation paradigms it has been shown that babies as old as 6 months are able to distinguish between arrays of items or sequences of sounds of 4 from 8, and 8 from 16 (ratio 1:2) (Lipton & Spelke, 2003; Xu & Spelke, 2000). Older babies can discriminate between finer ratios. At 9 months for example, babies can discriminate between displays of 8 and 12 items (ratio 2:3) (Lipton & Spelke, 2003) and between the age of 3 and 6 years, children can distinguish between ratios of 3:4 and 5:6 (Halberda & Feigenson, 2008). In adulthood, estimation skills peak, allowing discrimination between arrays with ratios of 9:10 (Halberda, Mazzocco, & Feigenson, 2008; Pica et al., 2004).

Such evidence from animal and infant studies suggests that basic estimation skills involved in number sense are evolutionarily conserved. However, this does not imply that individual differences in number sense are genetic in origin. Behavioural genetic studies have shown that in almost every aspect of human behaviour and cognition, individual variation is a product of both, environmental and genetic influences (Plomin, DeFries, Knopik, & Neiderhiser, 2012). Genetic influences on individual differences in a trait in a particular population are called heritability. Therefore, heritability does not refer to the genetic effects on the presence of a function (e.g. human ability to learn new information), but to the proportion of the variance in this function (e.g., people have different learning capacities) that can be explained by variance in human DNA.

Evidence from both animals and humans suggests that for morphological traits (e.g. weight, body size, height), individual differences are under stronger genetic influence than for fitness-related traits (e.g. fertility, longevity) (Visscher, Hill, & Wray, 2008). In other words, for traits that have a clear positive end on a continuum (the healthier — the better) vs. no clear positive end (not the taller the better), evolution is less permissive of genetic variability. If number sense is of primary importance for survival for many species, it is more likely that genes will not play a large role in determining individual differences in this ability. A similar example is attachment – an important evolutionarily preserved trait in mammals – which shows low heritability, suggesting that individual differences in attachment are largely a product of environmental influences (Plomin et al., 2012). Because directional selection depletes additive genetic variance (genetic effects that add up across genes and are inherited from parent to offspring), traits subjected to selection pressure would be expected to show lower heritability. To date, nothing is known about the relative contribution of genetic and environmental factors to the substantial variability in numerosity discrimination documented by previous research, reviewed in the following section.

### 1.2 Individual differences in numerosity discrimination

One fundamental parameter in estimation skills, used to assess an individual's number sense acuity, is the ratio of the items in the arrays that are being compared. Discrimination of numerosities in animals, infant humans and adult humans follows the Weber Law (Halberda et al., 2008; Libertus & Brannon, 2009, 2010; Nieder & Miller, 2003, 2004; Pica et al., 2004). The Weber Law (Weber, 1834) describes the relationship between the magnitude of the stimulus appraised and the ability to detect ‘the just noticeable change’ in such magnitude. Judging whether a set has more items than another is difficult when the discrepancy between the two displays is small. According to the Weber Law, the threshold of the minimum difference that can be detected is equal to the difference between the numbers of items in the two sets (the increment in quantity) divided by the number of items in the smallest of the two sets. This threshold is indexed by the Weber Fraction. For example, if one can tell, without counting, which is the larger set between a display with 5 items and one with 7 (ratio 5:7), the Weber Fraction associated to the number sense acuity for that person is 0.4, [(7 − 5) / 5]. Previous research has found that numerosity estimation improves with practice (e.g., susceptible to sensory adaptation in the visual modality, Burr & Ross, 2008). Moreover, it improves with development: Weber Fraction associated with the smallest perceived ratio range from 1.0 at 6 months to 0.11 in adulthood (Halberda et al., 2008; Pica et al., 2004).

Individual differences in numerosity discrimination emerge early in life. Although at 6 months, infants can, on average, distinguish between ratios not finer than 1:2, one study showed that at this age babies already exhibit stable individual differences in numerosity discrimination. Further, the study found that individual differences in the ability to detect changes in numerosity at 6 months predicted this ability at 9 months independently of short-term memory skills (Libertus & Brannon, 2010). Individual differences in numerosity discrimination abilities were also detected in 3- to 4-year-olds, as well as, 6, 14 and 16-year olds (Halberda & Feigenson, 2008; Halberda, Ly, Wilmer, Naiman, & Germine, 2012; Halberda et al., 2008; Mazzocco, Feigenson, & Halberda, 2011). Number sense has been studied mainly in young children; however, a recent study surveyed number sense in over 10,000 individuals between 11 and 85 years old (Halberda et al., 2012). The study reported individual differences and developmental changes in numerosity discrimination skills, identifying three main transitional age-related trends in the population: a rapid increase in discrimination accuracy between the age of 11 and 16, a steady improvement up the age of about 30, and a decline from 30 to 85.

It is possible that individual differences in numerosity estimation in children are driven by differences in the processing of perceptual characteristics of the stimuli rather than numerical information per se. Pre-school children have difficulties in ignoring continuous, non-numerical irrelevant information (e.g., the area occupied by the dots in display) in non-symbolic numerical comparisons (Rousselle & Noël, 2008). For example, when perceptual information was in conflict with numerical information (e.g., when arrays with more dots had smaller physical dot size than arrays with fewer dots), 4 year-olds were unable to discriminate between numerosities independent of the physical appearance of the stimuli (Soltész, Szücs, & Szücs, 2010). Moreover, discrepancies in results of studies of early number sense abilities may also stem from errors associated with difficulties of testing very young children.

Adults also seem to automatically process irrelevant non-numerical information (the area occupied by the dots for example) in numerosity discrimination, (e.g. Barth et al., 2006; Gebuis, Cohen Kadosh, de Haan, & Henik, 2009). Nevertheless, research has shown that numerosity information can be appreciated independently from physical attributes, such as texture and density (Ross & Burr, 2010) Empirical evidence suggests that in adulthood numerical information is as salient as the non-numerical (area) information, allowing responses to numerosity (discrete) rather than continuous properties of the stimulus (Nys & Content, 2012).

Whether individual differences in the processing of numerosity stem from perceptual processing of continuous or discrete information, accuracy in a simple task of judging which of two arrays has more items has been associated with mathematical abilities (e.g. Halberda et al., 2008; Lourenco, Bonny, Fernandez, & Rao, 2012; Mazzocco et al., 2011; Nys & Content, 2012). These studies indicate a positive association between accuracy in numerosity discrimination, mathematical performance and school achievement — across the life span (Halberda et al., 2012).

Research also suggests that numerosity discrimination is associated with measures of general cognitive ability. For example, the numerosity discrimination measure at age 16 in this study correlated with contemporaneous measures of mathematics (.33), speed of processing (.25), visuo-spatial working memory (.22), verbal and non-verbal ability (.19 and .27 respectively), language (.21), reading fluency and comprehension (.16). These relationships were explored using factor analysis. The measures are clustered into 3 factors: a verbal, a non-verbal and a perceptual dimension. Number sense loaded on the non-verbal factor, together with non-verbal ability, memory and mathematics and on the perceptual factor together with speed of processing — suggesting that variation in number sense is not only limited to variation in perceptual discrimination, but is also related to variance in other cognitive abilities. Further, after controlling for mathematics and a range of other cognitive abilities, numerosity discrimination significantly correlated with speed of processing (results are available from the authors). In the same sample, correlations of number sense (measured at age 16) with non-verbal ability, measured at different ages, remained significant (~ .15), even after controlling for longitudinal measures of mathematics. An association between numerosity discrimination and memory was found in a study of 4 year-old children (Soltész et al., 2010). Further investigations are required to explore the etiology of specificity and generality of the associations between numerical discrimination and other cognitive and learning abilities. One of the first steps towards understanding the nature of these associations is to explore the etiology of individual differences in number sense skills.

### 1.3 Hypotheses

The present study is the first large scale genetic investigation into the etiology of individual differences in number sense. We assessed number sense in 16-year-old twins and conducted genetic analyses in order to estimate the relative contribution of genetic and environmental factors to variation in number sense. The large and representative sample, which included both same-sex and opposite-sex twin pairs, also allowed the investigation of any sex differences in the etiology of the variation in number sense.

Given the association of number sense with mathematical and other cognitive abilities, for which moderate to high heritabilities have been shown by previous research (e.g., Kovas, Haworth, Dale, & Plomin, 2007; Plomin et al., 2012), it could be expected that heritability of number sense would be at least moderate. On the other hand, the ability to judge more from less may have developed as crucial for survival (e.g. through importance for obtaining food resources or judging danger), and therefore may be a product of ‘directional’ evolutionary selection. As mentioned earlier, such directional selection would reduce the frequency of genetic variants, leading to reduced trait-relevant genetic variation in subsequent generations (Plomin et al., 2012; Visscher et al., 2008). By this account, a more modest genetic contribution to individual differences in number sense than is usually seen for cognitive abilities may be expected.

In addition to estimating heritability of number sense using the twin method, we used Genome-wide Complex Trait Analysis (GCTA), to estimate heritability directly from DNA using 1.7 million DNA markers available for 1118 unrelated individuals in our sample (Yang, Lee, Goddard, & Visscher, 2011; Yang et al., 2010).

## 2 Methods

### 2.1 Sample

Twins Early Development Study (TEDS) is a large longitudinal study of twins born in England and Wales in 1994, 1995 and 1996 (Haworth, Davis, & Plomin, 2013). The sample has been shown to be representative of the UK population in terms of ethnicity, parental education and socio-economic status (Kovas, Haworth, Dale, et al., 2007).

The analyses for this investigation were carried out on the data collected from 3799 twin pairs of the 1994 and 1995 birth cohorts of TEDS when the twins were 16 years old. For the purpose of this study, twins with specific medical problems and whose English was not the first language were excluded from the analyses. The final sample consisted of 4518 twins (2259 pairs): 836 monozygotic (MZ), 733 dizygotic same-sex (DZss), and 689 dizygotic opposite-sex (DZos) pairs. The mean age for the sample was 16.6 years (SD = .28).

### 2.2 Measures and procedure

Since the wave of testing at age 10, TEDS' assessments have been mainly conducted via the Internet as it provides a cost-effective, quick and reliable method to collect data in such a large and widespread sample. The advantages and disadvantages of Internet testing have been reviewed in Birnbaum (2004) and more specifically for the TEDS sample in Kovas, Haworth, Dale, et al. (2007). The twins' families received by post an information pack about the study and log-ins to access the website for online testing. The twins' log-ins were activated after parents logged in and gave their consent online. Upon completing the tests, the twins were rewarded with a £10 shopping voucher and an entry into a prize draw.

The *Number Sense Task*, that assessed the ability to discriminate numerosities, was embedded in the web-based battery of the TEDS assessment at age 16. The battery could be accessed online at the TEDS website (www.teds.ac.uk) using a unique anonymized log-in. The task was an online implementation of the test described in Halberda et al. (2008), with some adjustment to the stimuli and parameters, in accordance to instructions provided by the author of the task. Prior to the online administration, the test was piloted for validity and suitability for the web-testing. More details on validity, reliability and a detailed description of the task are available from the authors. Briefly, the test consisted of 150 trials displaying arrays of yellow and blue dots, varying in size and with different numbers of dots of each colour. Each trial was presented for 400 ms; the task was to judge whether there were more yellow or blue dots. From the accuracy scores, a Weber Fraction was derived using least-squares method, as described in the supplementary information of Halberda et al. (2008). The correlation between the accuracy in the Number Sense Task and the derived Weber Fraction scores was .97 (p < .01, 2-tailed). In addition, reaction time was also recorded and used to correct scores so that the Weber Fraction for each participant was derived only on trials not considered outliers according to the Jolicoeur method (Van Selst & Jolicoeur, 1994). On average, 3.9 trials were removed from each performance, with a minimum of 0 and a maximum of 10 removed. The test included online instructions and practice trials and could be completed by the 16-year-old participants without supervision.

### 2.3 Twin analyses

Standard quantitative genetic analyses were used to estimate genetic and environmental influences on individual differences in number sense (Plomin et al., 2012). Similarity on a trait within pairs of twins can be attributed to genetic influences – the effects of all the alleles at all gene loci that affect the trait – and shared (common) environmental factors. All non-genetic influences that do not contribute to make the twins similar to one another are referred to as non-shared environments. If a trait is totally influenced by genetic factors, the monozygotic (MZ) twin correlation on that trait should be 1 because MZ twins are genetically identical, and the DZ correlation should be half of the MZ correlation because DZ twins, like any siblings, share on average only half of their variable DNA. If the correlation for MZ twins is less than 1, this is due to the influences of non-shared environment. If the DZ correlation is more than half of the correlation displayed by MZ twins, this increase in similarity is attributed to shared environmental factors. In twin methodology it is assumed that shared environmental factors are the same for MZ and DZ twins (Rijsdijk & Sham, 2002).

Analyses of twin data are carried out on the residuals of standardized scores corrected for average effects of age and sex (McGue & Bouchard, 1984). This is because twins' age across pairs is completely correlated, which could inflate twin correlations and be wrongly attributed to shared environmental influences. The same applies to sex because MZ co-twins are all of the same sex, as are half of DZ pairs.

### 2.4 Model-fitting analyses

Although the results of twin analyses can easily be gleaned from the simple twin correlations, structural equation model-fitting tests alternative models and provides confidence intervals for estimates of the proportion of variance within a trait that can be attributed to genetic (A), shared (C) and non-shared (E) environmental influences. In twin model fitting, the estimate for non-shared environment incorporates the measurement error — as unsystematic error can only contribute to the twins' dissimilarity in the measured trait. We employed standard model-fitting procedures. Parameters were estimated using OpenMx software (Boker et al., 2011) conducted in the R environment (http://www.R-project.org; R Development Core Team, 2011). In order to fit the most parsimonious model describing the data with the fewest number of parameters, simpler nested models were tested by dropping parameters. To determine the model that best fits the data, the fit of the nested models was compared against the fit of the full ACE model. The significance of the fit was evaluated from the difference in likelihoods between the full ACE model and the reduced model; significant p-values indicate that the reduced nested models fit less well than the full ACE model. The Akaike information criterion (AIC) and Bayesian information criterion (BIC) were also used to provide information about the goodness of the fit of the models: The lowest AIC and BIC refer to the most parsimonious (preferred) model.

### 2.5 Sex-Limitation model

Alternate models can be used to estimate two types of sex differences in the A, C and E parameters (Neale & Maes, 2003). Quantitative sex differences refer to differences in A, C, or E estimates for males and females. Qualitative sex differences rest on comparisons between same-sex and opposite-sex DZ twins, and indicate the extent to which the same genetic or environmental factors affect individual differences for males and females. It should be noted that quantitative and qualitative sex differences in the etiology of individual differences are unrelated to any observed mean sex difference.

To investigate qualitative and quantitative gender differences, ACE parameters and their 95% confidence intervals were estimated with the data divided into MZ male (MZm), MZ female (MZf), DZ male (DZm), DZ female (DZF), and DZ opposite-sex (DZos) twin pairs. Quantitative sex differences are tested by running a model that estimates A, C, and E parameters separately for males and females, this full model is then compared with reduced models that equate A, C, and E parameters for males and females. Qualitative sex differences are tested by comparing the variance and covariance for DZos and same-sex DZ (DZss). The genetic relatedness coefficient (r*_g_*) for DZ same-sex pairs (male and female) is 0.5 as DZ twins share half of their segregating genes. If different genes affect males and females, r*_g_* for DZos will be less than 0.5. If sex differences are quantitative, the same genetic factors influence males and females, therefore the r*_g_* for DZos will be 0.5, but A, C and E estimates for males and females will be significantly different. The same logic applies to the coefficient indicating relatedness due to shared environmental factors (r*_c_*), which should be equal to 1 as twins in the same family share the same environments. It is not possible to estimate r*_g_* and r*_c_* at the same time (the model is not statistically defined), so qualitative and quantitative differences in genetic influences have to be modelled separately from shared environmental influences.

Four models were fitted to the number sense raw data and, in order to determine which model described best the observed data, their fit was compared using the same criteria described for the estimation of the univariate ACE parameters. In the Full Sex-Limitation model, all the parameters were estimated separately in males and females, allowing for quantitative difference. The r*_g_* coefficient was also estimated to allow for qualitative sex differences. In the Common Effects Sex-Limitation model, the A, C and E parameters were estimated separately for males and females but the r*_g_* of the DZos was constrained to 0.5 thus allowing only for quantitative differences. If this model yielded a better fit compared to the Full model, quantitative but not qualitative differences between males and females would be indicated. The Scalar Effects Sex-Limitation model tested for variance differences between males and females. The A, C and E parameters were constrained to be the same for males and females, and r*_g_* was constrained to 0.5 in the DZos. In the Null Model, all the parameters were constrained to be the same for males and females, thus testing the null hypothesis that there are no etiological or phenotypic variance differences in number sense between males and females.

### 2.6 Genome-wide Complex Trait Analysis (GCTA)

GCTA can be used to estimate genetic variance accounted for by all the SNPs genotyped in samples consisting of unrelated individuals free of assumptions of the twin method or (Lee, Wray, Goddard, & Visscher, 2011; Yang, Lee, et al., 2011; Yang, Manolio, et al., 2011). GCTA requires large samples in which each individual has been genotyped for hundreds of thousands of DNA markers, typically single nucleotide polymorphisms (SNPs). Large samples and extensive genotyping are also needed in genome-wide association (GWA) studies, thus data collected in GWA studies are suitable to conduct GCTA analyses. GWA genotyping data of the TEDS sample have been used to conduct the first GWA studies of general cognitive abilities, mathematics and reading (e.g. Docherty et al., 2010; Haworth, Meaburn, Harlaar, & Plomin, 2007), as well as to conduct the first GCTA studies of cognitive abilities at the age of 12, estimating heritability between 20% and 35% for diverse cognitive abilities (Plomin et al., 2013). GCTA has been used to estimate heritability as captured by genotyping arrays for height (Yang et al., 2010), weight (Yang, Manolio, et al., 2011), psychiatric and other medical disorders (Lee et al., 2011; Lubke et al., 2012), and personality (Vinkhuyzen et al., 2012). GCTA has also been applied to general cognitive ability in studies of adults (Chabris et al., 2012; Davies et al., 2011) and children (Deary et al., 2012).

At age 16, GWA genotyping data and number sense data were available on about 1000 individuals in TEDS. Because GWA analysis needs to correct for multiple testing of hundreds of thousands of genotyping tests, these data are not suitable for GWA analysis, but they can be used in GCTA analyses to estimate genetic influence as a check on the heritability estimate based on the twin method.

In contrast to GWA which attempts to identify particular SNPs associated with a trait, GCTA uses chance similarity across hundreds of thousands of SNPs in a random effects model to predict phenotypic similarity pair by pair in a large sample of unrelated individuals. The essence of GCTA is to estimate genetic influence on a trait by predicting phenotypic similarity for each pair of individuals in the sample from their total SNP similarity. In contrast to the twin method, which estimates heritability by comparing phenotypic similarity of MZ and DZ twin pairs whose genetic similarity is roughly 100% and 50% respectively, GCTA relies on comparisons of pairs of individuals whose genetic similarity varies from 0 to 2%. GCTA extracts this tiny genetic signal from the noise of hundreds of thousands of DNA markers (single nucleotide polymorphisms, SNPs) using the massive information available from a large sample of individuals, each compared pair by pair with every other individual in the sample.

GCTA genetic similarity is not only limited to the additive effects of genotyped SNPs themselves but also includes unknown causal variants to the extent that they are correlated with the SNPs. Mendel's second law of inheritance is that genes (as we now call them) are inherited independently (now called linkage equilibrium), but Mendel did not know that genes can be on the same chromosome, in which case they are not inherited independently (linkage disequilibrium). This violation of Mendel's second law is complicated by the fact that during meiosis, chromosomes from the mother and father recombine on average once per meiosis, which means that, in the population, genes on the same chromosome are separated by this process of recombination to the extent that the genes are not close together on the chromosome. GCTA provides a lower-limit estimate of heritability because it misses genetic influence due to causal variants that are not highly correlated with the common SNPs on genotyping arrays.

Genetic effects on a trait may not just derive from the simple sum of independent genetic actions, they may stem from more complex gene–gene interactions. One of the assumptions of the twin method is that the variance explained by genetic influences is attributed to additive genetic effects. In practice, the method captures both additive and non-additive genetic effects because the DNA sequence of identical twins is virtually identical and thus they share all genetic effects including non-additive effects (see Plomin et al., 2012, for details). Conversely GCTA adds up the effect of each SNP, therefore it does not include gene–gene interaction effects; this is why the method provides lower-limit estimates of heritability caused by to additive genetic effects.

Genotyping on the Affymetrix 6.0 GeneChip and subsequent quality control was carried out as part of the WTCCC2 project (The UK IBD Genetics Consortium & the Wellcome Trust Case Control Consortium; Barrett et al., 2009) for 1118 individuals (one member of a twin pair) for whom number sense data at age 16 were also available. In addition to nearly 700,000 genotyped single-nucleotide polymorphisms (SNPs), more than one million other SNPs were imputed using IMPUTE v.2 software (Howie, Donnelly, & Marchini, 2009). GCTA estimates were obtained using the GCTA software package (Yang, Lee, et al., 2011). In GCTA, any pairs whose genetic similarity exceeded +/− 0.025 (i.e. greater genetic relatedness than fourth-degree relatives) are removed so that genetic similarity is random and can be treated in a random effects model. By this criterion, no individuals were excluded.

## 3 Results

**Fig. 1:**
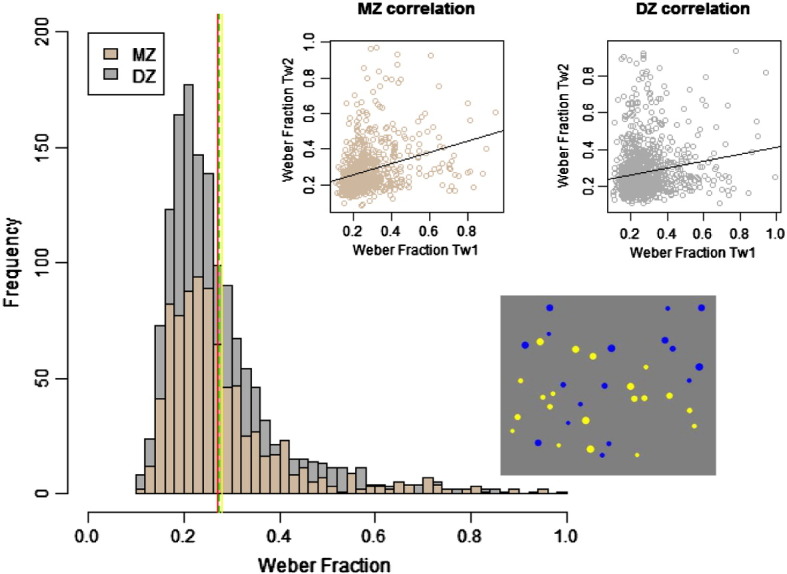
*S*catter plot correlations MZ (monozygotic, in brown) and DZ (dizygotic, in grey) twins with their co-twins for Weber Fraction raw scores. The Weber Fraction scores were derived from accuracy in the Number Sense Task. The display of yellow and blue dots is an example of a test trial. The twins had to judge whether there were more yellow or blue dots following an exposure of 400 milliseconds. The overlapping distributions of the Weber Fraction scores of the MZ (brown) and DZ twins (grey) show the means: MZ = .28 (green dashed line); DZ = .27 (red line). These are compared with the 16-year olds means reported in Halberda et al. (2012) = ~ .285 (yellow line).

The analyses were conducted using Weber Fraction and accuracy scores on the Number Sense Task. Prior to quantitative genetic analyses, accuracy scores were squared and a square-root transformation was applied to Weber Fraction scores. The variables were then standardized (mean of zero and standard deviation of one), corrected for age and sex and scores outside +/− 3 standard deviations were considered as outliers and excluded. The transformation improved normality of both variables. However, even after transformation, Weber Fraction scores did not fully meet assumptions of normality as skewness was 1.09 (SE = .05) and kurtosis 1.27 (SE = .10). Although maximum likelihood estimation assumes normality of the data, the method has been shown to be robust when assumptions of normality are violated (c.f. Boomsma & Hoogland, 2001). We also report the results of analyses conducted on the accuracy in the Number Sense Task which was skewed negatively (− .43; SE = .05) but not kurtotic (− .02; SE = .10). Number Sense accuracy showed good internal consistency (alpha = .79) and test–retest reliability (.62). Further information on the psychometrics of the measures is available from the authors. Descriptive statistics of the data collected on the TEDS sample are also consistent with results reported for 16-year-olds in Halberda et al. (2012) (see Fig. 1 for a comparison of means).

Table 1 shows means, standard deviations and ANOVA result by sex and zygosity for Number Sense accuracy and Weber Fraction scores. These descriptive statistics are reported for one twin chosen at random from each pair (N = 2259). Mean accuracy score on untransformed Number Sense accuracy was 115.82 (SD = 9.57; range = 79–140, out of a possible 150). Mean on the untransformed Weber Fraction score was 0.28 (SD = .13; range .10–.99). No significant mean sex differences were found, nor were there zygosity differences.

Descriptive analyses run on the other half of the sample yielded highly similar results (available from the authors).

Table 2 shows the intraclass correlations (indexing the similarity of co-twins) with 95% confidence intervals. Despite the reasonable validity of our task, the intraclass correlations for both measures of number sense were modest, even for MZ twins, suggesting that twins differ markedly in their number sense ability and pointing to a significant contribution of non-shared environmental influences. Nonetheless, MZ twin correlations were greater than DZ correlations, suggesting the presence of some genetic influence on number sense as well.

The model-fitting results confirmed these interpretations (Table 3). For both measures of number sense, the best fitting model included only genetic influence (A) and non-shared environmental influence (E).

As shown in Table 4, genetic influence was modest for both accuracy (.35) and for Weber Fraction (.32), with non-significant shared environmental influence. The rest of the variance was attributed to non-shared environment which also includes error of measurement.

The results of the Sex-Limitation model fitting are shown in Table 5. No quantitative or qualitative differences were found for Number Sense accuracy or the Weber Fraction. The models testing for qualitative and quantitative differences in both number sense variables (respectively the Common Effects and Scalar Effects models) did not differ significantly from the Full Sex-Limitation model. The AIC and BIC parameters confirmed that the best fit was provided by the Null Model, indicating that there are no qualitative or quantitative differences in the etiology of number sense between males and females. Genetic and environmental influences were estimated separately for males and females by fitting a Full Sex-Limitation model. The parameters for the accuracy and the Weber Fraction scores with their 95% confidence intervals are shown in Table 4.

Genome-wide Complex Trait Analysis (GCTA) yielded a non-significant estimate of zero heritability for number sense. Because the sample size was relatively small for GCTA analysis, the standard error of estimate was large (.29). Nonetheless, this GCTA analysis provides support for the relatively low twin study estimate of heritability for number sense.

## 4 Discussion

We performed the first large-scale genetically sensitive analysis on number sense and found that individual differences in this ability at age 16, as indexed by a measure of accuracy in numerosity discrimination and by the Weber Fraction, were only modestly influenced by genetic factors. Most of the variance was explained by non-shared environment (.68 for Weber Fraction and .65 for the accuracy scores). The modest estimate of heritability from the twin study was supported by a zero heritability estimate from the GCTA analysis. Because GCTA estimates are limited to the additive effects of common SNPs included on DNA arrays, GCTA estimates are typically about half of the estimates from twin studies. For example, another TEDS study estimated heritabilities for verbal ability as .40 and .26 for twin and GCTA analyses, respectively (Plomin et al., 2013).

As number sense is linked to other cognitive abilities, which have been found to be at least moderately heritable, its modest heritability may come as a surprise. However, as indicated earlier, evolutionarily useful traits are not necessarily heritable. Fear for example is considered an evolutionary useful trait; but individual differences in acquisition, habituation and extinction of fear in the presence of stimuli such as snakes and spiders, are mostly explained by environmental influences (Hettema, Annas, Neale, Kendler, & Fredrikson, 2003). In terms of genetic influences on evolutionarily preserved traits, such as number sense, one set of genes may provide a blueprint for the development of this ability across many species; whereas a different set of genes may contribute to variation in the trait between individuals in any population. Such ‘individual differences’ genes may work through various mechanisms, affecting for example perceptual processes, speed of processing, and other cognitive functions relevant to perform estimation of numerosities.

Heritability is a descriptive statistic specific to a particular age and population (Plomin et al., 2012). For this reason, we cannot generalize the heritability of number sense at age 16 in our UK sample to other ages or other populations. For example, reading abilities show consistent genetic and environmental estimates across ages and across populations (Byrne et al., 2007, 2005; Stromswold, 2001), while the heritability of general cognitive ability increases from early age to young adulthood (Davis, Haworth, & Plomin, 2009; Haworth, Dale, & Plomin, 2009). Similarly, we cannot exclude the possibility of developmental changes in the heritability of number sense. It is possible that the marked individual differences in number sense acuity observed in infancy (Libertus & Brannon, 2010) may be under stronger influence of genetic variation. This could explain why during infancy babies already show individual differences in discrimination of numerosities. However, later in development, factors such as exposure to numerical stimuli, individual's interest in numeric information, and amount of practice with number-related activities may all contribute to the development of this ability. With number sense becoming increasingly precise during development, individual differences in this precision may be under greater environmental influence. Since this study is the first large scale genetically sensitive investigation on number sense, further research needs to be conducted using longitudinal twin samples assessing etiological change and continuity of influences on number sense. In addition, the strong non-shared environmental influences indicated in this study call for cross-cultural genetically-sensitive investigations to examine the relative contributions of genes and environments to number sense in different cultures, where different educational, linguistic, and social practices operate.

One of the implications of the large environmental component of individual differences is that higher levels of accuracy in estimation of numerosity may be achieved through training or less focused experience. One study involving 6 month-old infants showed that when babies were simultaneously presented with a congruent visual and auditory stimulus they were able to discriminate numerosities with a ratio usually present in 9 month-old infants (Jordan, Suanda, & Brannon, 2008). One explanation given by the authors was that the greater amount of numerical information received in two rather than one sensory modality increases infants' arousal leading to increased sensitivity to numerical changes. Number sense in animals seems also to be influenced by external cues in the same way as in humans. In one study, fish learned to discriminate numerosity faster if the numerical information was available in more than one sensory source, suggesting that multisensory numerical information facilitates discrimination learning (Agrillo et al., 2011). It is important to remember that the estimates of genetic and environmental influences derived from the twin studies reflect ‘what is’, rather than ‘what could be’ or ‘what should be’. The finding that multisensory exposure improves numerical processing in the laboratory setting does not mean that individual differences in such exposure contribute to the observed variation in number sense development in the population. More research is needed in order to identify specific sources of such environmental influences.

Although we need to understand in more depth the mechanisms through which the environment acts upon numerosity discrimination skills, there are some studies showing how estimation of numerosity skills can be manipulated through exposure to numerical material. It has been suggested that playing numerical board games gives children familiarity about numbers and improves their estimation of numerical magnitudes (Siegler & Ramani, 2008, 2009). However, it is not clear why such influences should be non-shared by twins in the same family. It is possible that active and evocative gene-environment correlations, by which children choose specific activities or receive specific environmental inputs partly based on their genetic predispositions, play a role. Future studies should examine the similarity in twin and non-twin siblings in the willingness and frequency of engagement in the relevant activities — to evaluate whether they can explain some of the non-shared environmental influences on number sense development. Most importantly, such studies need to involve genetically sensitive designs to control for genetic influence in understanding the environment.

Studies on artificial learning provide further evidence that individual differences in numerosity skills similar to number sense can be taught. Neural network models can be modelled to detect numerosity from visual inputs (Domijan, 2004), with the quality of detection depending on the quality (e.g. frequency) of the inputs. One study has shown that models not programmed a priori in numerosity recognition can learn to discriminate numerosities according to the Weber Law through ‘unsupervised learning’ (Stoianov & Zorzi, 2012). The model in the study was also able to simulate response to numerosities similarly to the neurons in the areas responsible for numerosity representation (later intraparietal area) of the human (Santens, Roggeman, Fias, & Verguts, 2010) and monkey brain (Roitman, Brannon, & Platt, 2007). As it is possible for models to develop different levels of number sense just by being exposed to different qualities of visual stimuli, humans could develop differences in number sense through different exposures to numerical material — as opposed to genetic influences setting individual differences (programs in the case of the models).

Our results add a novel perspective on a current debate in the mathematical literature. One theory proposes that the severe mathematical disability of Developmental Dyscalculia emerges from difficulties in numerosity processing. This occurs even in the absence of general cognitive impairments (Butterworth, 2005; Landerl et al., 2004). It has been suggested that this problem with basic numerosity manipulation may be genetic in origin (Butterworth, 2005). Indeed, although multivariate genetic research suggests that individual differences in mathematical ability and disability are largely influenced by the same genetic factors as those that affect other learning and cognitive traits, some unique genetic effects also exist (Kovas, Haworth, Petrill, & Plomin, 2007). These unique genetic effects could be those shared between number sense and mathematics.

Evidence shows that variation in number sense may also arise under the influences of general cognitive development (e.g. Geary, 2011; Geary, Hoard, Nugent, & Byrd-Craven, 2008; Swanson & Sachse-Lee, 2001). It is possible that children with poor reading, poor memory, or low general cognitive ability engage in less effective or insufficient numerical practices (e. g. less games with numerical content during pre-school age) compared to children with non-impaired general abilities. In the long term, these differences in numerically-relevant environments may lead to the observed differences in numerosity processing. In other words, it is possible that variation in numerosity discrimination may be a product, rather than a cause of mathematical or general cognitive ability variation. Alternatively, the same etiological factors could affect the traits without any reciprocal contributions between them.

Studies on number sense have reported no mean sex differences in this ability, with the exception of one study that revealed marginal male advantage in numerosity discrimination in 4 year-old children (Soltész et al., 2010). As mentioned earlier, the etiology of average differences may be independent from the etiology of variation. The present study was the first to examine whether the etiology of individual differences in number sense was the same for males and females. We found no quantitative, qualitative or variance sex differences in numerosity discrimination skills. In other words, factors that make males differ from one another in number sense are the same that make females differ from one another. Because of the close relationship between numerosity discrimination and mathematical ability, it needs to be noted that earlier quantitative genetic investigations have found no sex differences in the etiology of different aspects of mathematical abilities, disabilities, or high abilities. This indicates that same genetic and environmental factors affect individual differences in mathematics equally in males and females (Kovas, Haworth, Dale, et al., 2007; Kovas, Haworth, Petrill, et al., 2007; Markowitz, Willemsen, Trumbetta, van Beijsterveldt, & Boomsma, 2005; Petrill, Kovas, Hart, Thompson, & Plomin, 2009). The absence of sex differences in numerosity discrimination skills suggests that any observed average sex differences in mathematics (e.g. Leahey & Guo, 2001; Penner & Paret, 2008) are not mediated by estimation of numerosity skills, at least at the age of 16.

## 5 Conclusion

The two methods employed in this study, the twin method and the GCTA analysis, showed that individual differences in numerosity estimation are only modestly influenced by genetic factors. One interpretation of these results is that number sense has evolved as crucial for survival (Panteleeva, Reznikova, & Vygonyailova, 2013). Similar to other traits undergoing directional natural selection, disadvantageous alleles may have been selected against, leading to reduced additive genetic variability. Other factors could contribute to the low heritability, including, potential non-additive genetic effects (not picked up by the GCTA analyses) or the issues of measurement.

Sex differences in number sense are minimal, both descriptively and etiologically: in addition to finding no mean sex difference, we also find that the same genetic factors influence individual differences in number sense skills in males and females equally.

As number sense measured at 16 is associated with general intelligence, we plan to investigate the etiology of the links between number sense, general intelligence, and other cognitive abilities such as spatial ability and other learning abilities such as mathematics. As the environment is a major source of individual variation in number sense, it is particularly important to understand its role in the covariation among these traits and to identify specific environmental factors involved.

## Disclosure statement

The authors have declared that no competing interests exist.

## Figures and Tables

**Table 1:** Means, standard deviations and ANOVA results by sex and zygosity.

																							ANOVA						
Measures	All		MZ		DZ		Female		Male		MZm		DZm		MZf		DZf		DZo		DZss		Zyg.		Sex		Zyg. ∗ Sex		Tot.
	M	SD	M	SD	M	SD	M	SD	M	SD	M	SD	M	SD	M	SD	M	SD	M	SD	M	SD	p	η²	p	η²	p	η²	R2
Number Sense accuracy	N = 2258		N = 836		N = 1422		N = 1315		N = 943		N = 317		N = 626		N = 519		N = 796		N = 689		N = 733								
	.03	.98	.00	.99	.04	.98	.05	.95	.00	1.0	− .07	1.1	.04	1.0	.04	.93	.05	.97	.04	.99	.05	.98	.17	.00	.16	.00	.29	.00	.000

Weber Fraction	N = 2214		N = 817		N = 1397		N = 1298		N = 916		N = 301		N = 615		N = 516		N = 782		N = 677		N = 720								
	− .10	.85	− .07	.85	− .11	.84	− .10	.83	− .10	.87	− .08	.86	− .10	.88	− .07	.85	− .12	.82	− .10	.85	− .12	.83	.34	.00	.95	.00	.79	.00	.001

Number Sense accuracy = accuracy scores on Dot Task (squared transformation); Weber Fraction = Weber Fraction score (square root transformed); M = mean; SD = standard deviation; MZ = monozygotic twins; DZ = dizygotic twins; MZm = monozygotic males; MZf = monozygotic females; DZo = dizygotic opposite sex; DZss = dizygotic same sex; p = p-value associated with the effect size of sex, zygosity and the interaction of the two on the means of all groups; η^2^ = magnitude of the effect of sex, zygosity and the interaction of the two on the means of all groups; R^2^ = proportion of variance explained by sex and zygosity; N = number of twins: one randomly selected from each pair. Scores outside +/− 3 standard deviations have been removed. The standardized means and standard deviations show that both variables are not normally distributed. Accuracy scores on the Number Sense Task are less skewed compared to the Weber Fraction scores.

**Table 2:** Intraclass correlations for MZ and DZ twins.

Measure	r MZ (N) (95%CI)	r DZ (N) (95%CI)
Number Sense accuracy	.35 (730)	.18 (1175)
	(.28–.41)	(.13–.24)
Weber Fraction	.31 (700)	.15 (1140)
	(.24–.38)	(.09–.20)

rMZ = intraclass correlation for monozygotic twins; rDZ = intraclass correlation for dizygotic twins; N = number of complete pairs; 95% CI = 95% confidence intervals.

**Table 3:** Univariate model-fitting results.

Measure	Model	− 2LL	df	(Δ − 2LL)	AIC	(Δ − AIC)	BIC	p-Value	p
Number Sense accuracy	Saturated	− 12,649.05	4505		3639.05		− 12,009.57	–	10
	ACE	− 12,658.33	4511	− 9.28	3636.33	2.72	− 12,029.34	.10	4
	**AE**	**− 12,658.58**	**4512**	**− .25**	**3634.58**	**1.75**	**− 12,033.29**	**.60**	**3**
	E	− 12,791.82	4513	− 133.49	3765.82	− 129.49	− 11,970.74	.00	1
Weber Fraction	Saturated	− 11,170.54	4415		2340.54		− 12,382.55	–	10
	ACE	− 11,185.37	4421	− 14.83	2343.37	− 2.83	− 12,399.55	.02	4
	**AE**	**− 11,185.37**	**4422**	**.00**	**2341.37**	**2**	**− 12,403.62**	**1.0**	**3**
	E	− 11,282.29	4423	− 96.92	2436.29	− 92.92	− 12,359.23	.00	1

− 2LL = minus log-likelihood; df = degrees of freedom; Δ − 2LL = difference in likelihood; AIC = Akaike's Information Criterion; Δ − AIC = difference in AIC, this is calculated between the Saturated and full ACE model, and between the full ACE model and the AE and E nested models. BIC = Bayesian Information Criterion; p-value = associated with the differences in likelihood ratio between the Saturated and the full ACE model, and between the full ACE model and the AE and E nested models. p = number of parameters estimated. The p-value shows no significant differences in likelihood between the Saturated and the full ACE model for accuracy in the Number Sense Task scores. AIC shows good fit of the ACE model compared to the Saturated model in Number Sense scores (lower AIC of full ACE). The same parameter shows the better fit of the AE model. The goodness of fit for the Weber Fraction model is demonstrated to a lesser extent by the AIC and p-value. The BIC however shows a good fit of the full ACE model to the observed data and, similarly to the accuracy scores, confirms the best fit of the AE model for the Weber Fraction variable. The bold characters indicate the best fitting model.

**Table 4:** Parameter estimates for males and females separately and together.

Measure		Variance of A (95%CI)	Variance of C (95%CI)	Variance of E (95%CI)
Number Sense accuracy	Males	.35 (.07–.44)	.00 (.00–.24)	.65 (.56–.75)
	Females	.34 (.13–.41)	.00 (.00–.17)	.66 (.59–.74)
	**All**	**.35 (.30–.41)**	**.N/A**	**.65 (.59–.70)**
Weber Fraction	Males	.34 (.13–.43)	.00 (.00–.16)	.67 (.56–.77)
	Females	.29 (.06–.37)	.00 (.00–.19)	.71 (.63–.80)
	**All**	**.32 (.26–.37)**	**N/A**	**.68 (.63–.74)**

A, C, E = estimates respectively of genetic influences, shared environment, non-shared environment. 95% CI = 95% confidence intervals. Estimates separate for males and females and together for the accuracy scores and the Weber Fraction scores. The overlapping CI of the parameter estimates in males and females shows that the estimates of males and females do not significantly differ. Parameter estimates for males and females separately are from the Sex-Limitation model fitting. Estimates for males and females together are from the univariate model fitting, reported in bold font. The best fitting model did not include estimates for shared environment.

**Table 5:** Sex limitation model fitting results.

Measure	Model	− 2LL	df	(Δ − 2LL)	p-Value	AIC	BIC
Number Sense accuracy	Full Sex-Limitation model	10,791.89	3839	–	–	3113.89	− 9898.58
	Common.Eff. (Qualit. diff.)	10,793.70	3840	1.81	.18	3113.70	− 9901.66
	Scalar.Eff. (Quantit. diff.)	10,792.21	3842	.32	1.0	3108.21	− 9910.37
	**Null Model**	**10,794.07**	**3843**	**2.18**	**.70**	**3108.07**	**− 9913.43**
Weber Fraction	Full Sex-Limitation model	9533.15	3761	–	–	2011.15	− 20,434.40
	Common.Eff. (Qualit. diff.)	9533.3	3762	.154	.70	2009.30	− 20,442.21
	Scalar.Eff. (Quantit. diff.)	9533.79	3764	.492	.89	2005.79	− 20,457.66
	**Null Model**	**9533.99**	**3765**	**.842**	**.88**	**2003.99**	**− 20,465.43**

− 2LL = minus log-likelihood; df = degrees of freedom; Δ − 2LL = difference in likelihood; df = degrees of freedom; p-value = associated with the differences in likelihood ratio between each of the nested models and the Full Sex Limitation model. AIC = Akaike's Information Criterion; BIC = Bayesian Information Criterion. The models testing for qualitative and quantitative differences show no significant difference in fit compared to the full model (p-values non significant). The Null Model shows no significant difference in fit with the Full Sex-Limitation model suggesting no qualitative or quantitative differences, or variance differences between males and females in Number Sense Task accuracy scores and Weber Fraction. The bold font indicates the best fitting model.
